# Iron and Ferroptosis More than a Suspect: Beyond the Most Common Mechanisms of Neurodegeneration for New Therapeutic Approaches to Cognitive Decline and Dementia

**DOI:** 10.3390/ijms24119637

**Published:** 2023-06-01

**Authors:** Michele Cerasuolo, Irene Di Meo, Maria Chiara Auriemma, Francesca Trojsi, Maria Ida Maiorino, Mario Cirillo, Fabrizio Esposito, Rita Polito, Anna Maria Colangelo, Giuseppe Paolisso, Michele Papa, Maria Rosaria Rizzo

**Affiliations:** 1 Department of Advanced Medical and Surgical Sciences, University of Campania “Luigi Vanvitelli”, 80138 Naples, Italy; michele.cerasuolo@studenti.unicampania.it (M.C.); irene.dimeo@unicampania.it (I.D.M.); mariachiara.auriemma1@studenti.unicampania.it (M.C.A.); francesca.trojsi@unicampania.it (F.T.); mariaida.maiorino@unicampania.it (M.I.M.); mario.cirillo@unicampania.it (M.C.); fabrizio.esposito@unicampania.it (F.E.); giuseppe.paolisso@unicampania.it (G.P.); 2 Department of Clinical and Experimental Medicine, University of Foggia, 71122 Foggia, Italy; rita.polito@unifg.it; 3 Laboratory of Neuroscience “R. Levi-Montalcini”, Department of Biotechnology and Biosciences, NeuroMI Milan Center for Neuroscience, University of Milano-Bicocca, 20126 Milano, Italy; annamaria.colangelo@unimib.it; 4 Laboratory of Neuronal Networks Morphology and System Biology, Department of Mental and Physical Health and Preventive Medicine, University of Campania ‘‘Luigi Vanvitelli”, 80138 Naples, Italy; michele.papa@unicampania.it

**Keywords:** iron, ferroptosis, cognitive decline, Alzheimer’s disease, blood–brain barrier, reactive oxygen species (ROS), T2DM, chelators

## Abstract

Neurodegeneration is a multifactorial process that involves multiple mechanisms. Examples of neurodegenerative diseases are Parkinson’s disease, multiple sclerosis, Alzheimer’s disease, prion diseases such as Creutzfeldt–Jakob’s disease, and amyotrophic lateral sclerosis. These are progressive and irreversible pathologies, characterized by neuron vulnerability, loss of structure or function of neurons, and even neuron demise in the brain, leading to clinical, functional, and cognitive dysfunction and movement disorders. However, iron overload can cause neurodegeneration. Dysregulation of iron metabolism associated with cellular damage and oxidative stress is reported as a common event in several neurodegenerative diseases. Uncontrolled oxidation of membrane fatty acids triggers a programmed cell death involving iron, ROS, and ferroptosis, promoting cell death. In Alzheimer’s disease, the iron content in the brain is significantly increased in vulnerable regions, resulting in a lack of antioxidant defenses and mitochondrial alterations. Iron interacts with glucose metabolism reciprocally. Overall, iron metabolism and accumulation and ferroptosis play a significant role, particularly in the context of diabetes-induced cognitive decline. Iron chelators improve cognitive performance, meaning that brain iron metabolism control reduces neuronal ferroptosis, promising a novel therapeutic approach to cognitive impairment.

## 1. Introduction

In the neurodegenerative processes causing dementia, the prominent role is extracellular amyloid-beta (Aβ) plaques and intra-neuronal neurofibrillary tangle expression, synapse loss, and neuronal death, associated with progressive cortical and hippocampal neuronal dysfunction, respectively [1]. Several different causes, including genetic and epigenetic factors [2], or critical mechanisms, such as oxidative stress, mitochondrial dysfunctions, fragmentation of the Golgi apparatus, endoplasmic reticulum stress, a defect in protein synthesis and degradation (ubiquitin–proteasome system and autophagy), and failure of axonal transport [3,4], are other events involved in the neurodegenerative process, and neuronal cell death. More recently, some studies have associated iron accumulation to neurodegenerative processes, suggesting that iron plays a role in the development of several neurodegenerative diseases [5,6]. The normal range for serum iron is about 3–5 g; in particular, there are 35–45 mg of iron/kg of body weight, while in premenopausal women, the quantity is lower due to losses due to regular menstrual bleeding. In circulating red blood cells (RBCs), hemoglobin carries over two-thirds of the total iron (~1800 mg). In the main storage depots, such as the liver and reticuloendothelial macrophages, iron is present, respectively, for about 1000 mg and about 600 mg. The remaining iron (~10−15%) is found in the muscle fibers to release oxygen by myoglobin [7]. In total, 1–2 mg of dietary iron is absorbed daily with food [8,9], balanced by various losses that occur in the sloughing of intestinal mucosa cells [8,9] or menstruation in females [10].

When iron homeostasis is disrupted, iron accumulation can generate free radicals, resulting in high toxicity [11] responsible for metabolic diseases, such as non-alcoholic steatohepatitis, atherosclerosis, stroke, and type 2 diabetes [12], as well as for neurodegenerative diseases, such as Parkinson’s disease (PD) [13], multiple sclerosis (MS) [13], Alzheimer’s disease (AD) [6], prion diseases such as Creutzfeldt–Jakob’s disease (sCJD) [14], and amyotrophic lateral sclerosis (ALS) [13]. In addition, iron metabolism worsens in aging, causing iron accumulation, and affecting the inflammatory process, protein aggregation, and neuronal function [11].

Iron accumulating at toxic levels within neurons may lead to cell death via apoptosis, autophagy, necrosis, or ferroptosis, a recently discovered mechanism of iron-mediated cell death distinct from apoptosis [15].

Therefore, to better understand other possible mechanisms that can cause cognitive decline and dementia, we will consider the physiological brain iron role, the alteration of its homeostasis, and related brain interferences.

## 2. Iron Storage, Metabolism, and Function in the Brain

Iron is essential for the normal cellular functioning of the central nervous system (CNS). In the brain, the most preferred sites of iron are the pallidum, red nucleus, substantia nigra pars reticulata, Luy’s subthalamic nucleus, dentate nucleus of the cerebellum, and putamen. Frontal white matter shows a much more significant amount of iron than occipital [16]. Different CNS resident cells use iron. In neurons, iron is involved in many fundamental biological processes in the brain, including oxygen transportation, DNA synthesis, mitochondrial respiration, myelin synthesis, neurotransmitter synthesis, and metabolism. In oligodendrocytes, iron is mainly found as ferritin and transferrin. In astrocytes and microglia, the iron form remains undefined [13].

Iron transport and storage in the body is a complex process that involves several stages. Iron absorption occurs in the duodenum and upper jejunum and, once in the blood, iron is bound to the transport protein transferrin (Tf). Tf is synthesized almost exclusively in the liver and is secreted into the blood, but other tissues and organs, including the brain, also produce transferrin [17]. A small Tf amount would appear to be synthesized directly by oligodendrocytes [18].

Although the mechanisms for entering the brain are not fully understood, iron gets from the blood to the brain via the blood–brain barrier (BBB) [19]. There are two possible iron transport pathways: transferrin-bound iron (Tf-Fe) or non-transferrin-bound iron (NTBI) [20]. Due to the presence of transferrin receptor 1 (TfR1), highly expressed by neurons, the Tf/TfR1 pathway is the primary mechanism by which iron crosses the capillary endothelial membrane [21]. 

Further to the mechanism used to cross the BBB, iron can enter the brain through the epithelial cells of the choroid plexus [22,23,24], a structure characterized by fenestrated capillaries that allow the passage of Tf [23].

Subsequently, through the endocytic vesicles, iron crosses the cell, where the acidic environment facilitates the ferric ion release from Tf, transforming it into a ferrous ion by the endosomal reductase [22]. 

The following stages of transport still need to be well defined. The ferrous ion could be transported to the cytosol [25], stored in the form of cell ferritin [26], imported at the mitochondrial level [27], or released into the extracellular fluid [28]. Thus, when sequestered by ferritin, the level of free iron is reduced [29]. 

In turn, ferritin synthesis is increased by hypoxia in cortical neurons and decreased in glial cells [28] and the autophagy–lysosome system [30], resulting in increased iron release [31]. Whenever there is iron accumulation, the cerebrospinal fluid (CSF), in which iron saturation is almost 100%, has a low buffering capacity. Lastly, iron is exported by the protein ferroportin (FPN1), and transported into the blood by transferrin. In the presence of hepcidin, ferroportin is internalized [32]. 

Iron is the most abundant metal in neurons, contributes to 20% of energy consumption that occurs in the brain [33], and is vital for a number of cellular processes including neurotransmitter reactions essential to life—playing an important role in the brain [34]. Disruption of iron homeostasis can affect neurophysiological mechanisms, cognition, and social behavior, which eventually contributes to the development of neurodegenerative diseases [35].

## 3. Iron and Ferroptosis

**Figure 1:**
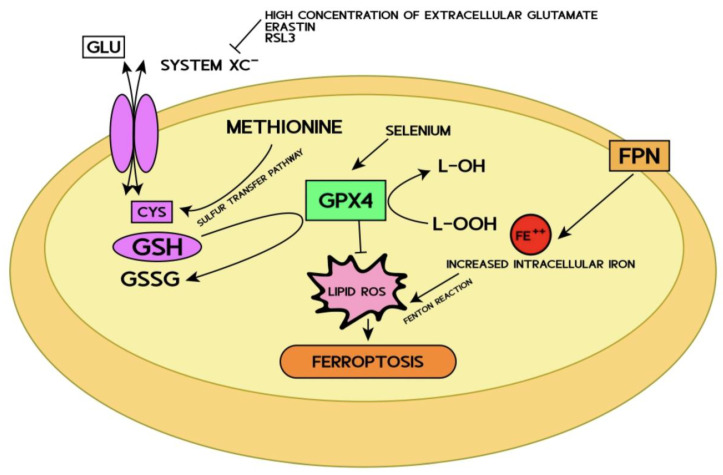
Schematic mechanisms of ferroptosis pathways.

Ferroptosis is a programmed cell death characterized by iron-dependent oxidative damage and subsequent plasma membrane rupture (Figure 1) starting from a redox imbalance between oxidants and antioxidants [15]. In normal cells, both iron and reactive oxygen species (ROS) are carefully managed by the cell itself. Free iron excess generates oxidative stress, particularly in the brain, where anti-oxidative defenses are relatively low [36].

Iron accumulation determines a progressive imbalance between the antioxidant defense mechanisms and the intracellular production of ROS [37]. ROS are chemically highly reactive molecules that contain oxygen and produced in all cell types due to metabolic activity. Mitochondria represent the main sites where ROS is present, as these organelles have redox transporters and enzyme complexes that can lose electrons, causing the formation of superoxide anions. Following this constant production of free radicals, the mitochondria contain, in their matrix, a specific efficient antioxidant system [38]. 

Many ROS are formed from hydrogen peroxide H_2_O_2_ and transition metals, and, among the various toxic species, the hydroxyl radical (OH·) and the peroxynitrite ion (ONOO-) are the free radicals most responsible for cell damage. Hydroxyl radicals trigger lipid peroxidation. Following this process, a loss of membrane permeability to calcium occurs with its associated cellular toxicity. Excessive production of ROS and the consequent imbalance of the redox state of the neuronal cell can cause damage determining the non-selective oxidation of proteins, carbohydrates, nucleic acids, and lipids [39,40]. It is very likely that uncontrolled lipid oxidation and the formation of lipid ROS lead to damage and perforation of the membrane [41,42].

While some studies argue that ROS production is unnecessary for ferroptosis [43], more recent studies have confirmed the need and contribution of mitochondrial ROS production, associated with DNA stress and metabolic reprogramming, in promoting lipid peroxidation with consequent induction of ferroptosis [44,45].

Although ferroptosis is morphologically, biochemically, and genetically distinct from apoptosis, necrosis, and autophagy, most studies concur that cells undergoing ferroptosis usually show necrosis-like morphological changes. Among these changes, we can mention the rounding up of cells, loss of cell membrane integrity, swelling of cytoplasmic organelles, chromatin condensation, and cytoplasmic swelling (oncosis) [46].

In mitochondrial cells, ferroptosis produces phenotypic change accompanied by increased membrane density, reduced or an absent ridge, outer membrane changes, and swelling [43,46,47].

Despite the evident morphological changes at the mitochondrial level, their role in the context of ferroptosis is still very controversial. Mitochondria, as previously reported, are the key element of cellular metabolism and are responsible for the production of ROS. It is unclear why only iron can induce ferroptosis compared to other metals. Iron activates specific downstream effectors that can trigger the ferroptosis mechanism after the production of ROS [48].

Although iron may directly generate excessive ROS through the Fenton reaction (Figure 1), there are classical ferroptosis activators such as erastin or RAS-selective (RSL3), which inhibit the antioxidant system favoring intracellular iron accumulation [43]. 

Erastin is a small molecule capable of initiating ferroptosis. Erastin and RSL3 inhibit cystine import via the cystine/glutamate antiporter system Xc- [43,49] required for exchange with intracellular glutamate. This transport mechanism is ATP-independent and is related to the high intracellular glutamate level. Additionally, high concentrations of extracellular glutamate inhibit the Xc- system [50]. Cystine transported into the cell becomes cysteine, used to synthesize proteins, and the non-ribosomal tripeptide antioxidant glutathione (GSH). Cysteine is the less abundant amino acid of the three composing the GSH, thus considered limiting for the de novo synthesis of GSH. Cysteine can be synthesized in some cells from methionine through the trans-sulphuration pathway (Figure 1). However, importing cystine appears critical in other cells to maintaining cysteine and GSH levels and preventing ferroptosis, at least in vitro [51]. Glutamate, RSL3, and erastin mediate GSH depletion, glutathione peroxidase 4 (GPX4) inhibition, and lipid peroxide formation [43,52,53].

GPX4 is the main ferroptosis regulator, a GSH-dependent enzyme that reduces lipid hydroperoxides (L-OOH) to lipid hydroxides (L-OH), which in turn can prevent the iron (Fe++)-dependent formation of toxic lipid ROS. At the same time, during the reduction process of L-OOH to L-OH, GSH is oxidized to GSSG, which is then converted into GSH by glutathione reductase (GRX) (Figure 1). GSH has a prominent role because it acts as a GPX4 cofactor maintaining high levels through the exchange of glutamate and cysteine via the cystine/glutamate antiporter system Xc-. Several ways regulate GPX4 activity: it can be upregulated by supplementing intracellular selenium or modulated through erastin, glutamate, and RSL3 (Figure 1). Inhibition of the system Xc-, inhibition of GSH synthesis, or direct inactivation of GPX4 will deplete GPX4, ultimately resulting in lipid peroxide accumulation and cell death due to iron overload, which sequentially catalyzes the production of toxic lipid ROS via the Fenton reaction [43,52,53].

Other conditions causing aberrant primary iron overload include inherited disorders of iron metabolism characterized by two main mechanisms: (1) the alteration of hepcidin–ferroportin interaction that induces increased intestinal iron absorption and macrophage iron release, leading to tissue iron overload; and (2) a defective expression of ferroportin on the cell membrane, due to loss-of-function mutation of SLC40A1 that impairs the iron export efficiency of ferroportin, causing iron retention in reticuloendothelial cells and hyperferritinemia with normal transferrin saturation [54].

Genetic iron overload, referred to as hereditary hemochromatosis (HH), includes five different genetic forms. The first mechanism leading to iron overload is the mutation in the high Fe++ (HFE) gene, which is involved in iron metabolism. HFE protein is expressed in choroid plexus epithelial cells, endothelial cells of the microvasculature, and ependymal cells lining the ventricle in the brain. Given the presence and location of the HFE protein at the interface between the brain and the vasculature and the cerebrospinal fluid (CSF), where it can influence brain iron uptake [54,55], it is not a surprise that the mutant forms of HFE could contribute to iron overload in neurodegenerative disorders.

There is also non-HFE hemochromatosis, a rarer form of HH. It has been demonstrated that mutations in HFE are associated with neurodegenerative diseases by increasing the production of free radicals in the brain, favoring neuroinflammation [54,55].

High concentrations of glutamate, erastin, and RSL3, blocking the Xc- system, inhibit cysteine entry into the cell by reducing the cellular levels of GSH, with consequent inhibition of GPX4 and the formation of lipid ROS, inducing ferroptosis.

Abbreviations: GLU: glutamate; GSH: glutathione; GSSG: glutathione disulfide; GPX4: glutathione peroxidase 4; RSL3: RAS-selective lethal 3; CYS: cysteine; L-OH: lipid hydroxide; L-OOH: lipid hydroperoxide; Fe++: ferrous iron; FPN: ferroportin.

## 4. Iron and Ferroptosis in Neurodegeneration

Growing evidence has indicated that iron deposition is one of the critical factors leading to neurodegeneration through ferroptosis and that iron-related oxidative stress is crucial in ferroptosis [56]. Although the correlation between altered iron homeostasis and neurodegeneration is well proven, all mechanisms are not fully understood. It is still unknown how the increased iron levels in the brain can produce oxidative stress and the inflammatory cascade generating maladaptive plasticity, neuronal death, and cognitive decline [57]. In addition, for example, in many neurodegenerative diseases, “advanced glycosylation end products” (AGEs) are increased. AGE products represent a marker of transition metal-induced oxidative stress and induce the formation of cross-links between proteins and ROS. Therefore, since such molecules could reflect early pathological changes rather than mere epiphenomena [58], it would be interesting to investigate them in the context of the ferroptosis process.

Several neurodegenerative diseases, such as Parkinson’s disease (PD), multiple sclerosis (MS), Alzheimer’s disease (AD), prion diseases such as Creutzfeldt–Jakob’s disease (sCJD), and amyotrophic lateral sclerosis (ALS), are characterized by the presence of common pathogenetic mechanisms producing neuronal death.

Parkinson’s disease (PD) is a degenerative and progressive motor disorder following the loss of dopaminergic neurons in the substantia nigra pars compacta. In PD, in addition to oxidative stress caused by several factors, including aging and neuroinflammation, excessive iron accumulation [59,60,61], GSH reduction, and GPX4 inhibition lead to ferroptosis, resulting in neurotoxicity [62]. Ferroptosis would therefore accelerate the progression of PD by interfering with the function of GPX4 [63]. Transgenic mice having a deletion of GPX4 show alterations and cognitive decline [64]. So, GPX4 is protective against neurodegeneration, also in PD, as an inhibitor of ferroptosis and with sustaining GSH function [65].

Some studies have reported conflicting results regarding dietary iron intake in subjects with PD. Hare et al. [66] pointed out that increased consumption of red meat may increase the risk of developing PD because red meat contains heme and thus increases intracellular iron concentration, producing ROS, metabolic alterations, and cellular damage. Instead, Miyake et al. [67] showed that dietary iron could have a neuroprotective effect to counteract the onset and progression of PD.

Multiple sclerosis (MS) is a chronic inflammatory demyelinating disease affecting the central nervous system and characterized by an acute or subacute onset of neurological symptoms such as loss of visual acuity and sensory or motor dysfunction. In multiple sclerosis, immune cells pass across the blood–brain barrier into the central nervous system, where they secrete pro-inflammatory cytokines and promote demyelination and axonal damage resulting from injury of oligodendrocytes [68].

In MS patients, iron distribution appears inhomogeneous. The iron content seems to be higher in the proximity of the lesions and at the level of the deep gray matter, while it appears to be reduced in the white matter [69]. The excess iron increases the production of ROS, resulting in the loss of myelin and neurons with consequent demyelination and neurodegeneration [70]. Studies in mouse models of multiple sclerosis have shown the association between iron overload in the central nervous system and the worsening of symptoms [71]. Sex-related differences were also found. In females, iron overload resulted in the early development of symptoms, while in males, the disease was more severe, with a higher mortality rate [72].

However, for oligodendrocytes that are known to have a high energy demand, iron represents an antioxidant mechanism to produce and maintain the myelin sheath [73,74]. 

Various studies have demonstrated that iron is also vital for developing oligodendrocyte progenitor cells (OPCs) into oligodendrocytes [73,74]. OPCs are recruited during the remyelination process from MS lesions and differentiate into mature oligodendrocytes.

Since many enzymes in these processes require iron, iron levels in oligodendrocytes are crucial for promoting remyelination and neuronal repair. Moreover, OPCs are much more sensitive than oligodendrocytes to the depletion of antioxidants, such as glutathione; therefore, they need higher antioxidant protection when inflammatory mediators and ROS increase. In this condition, iron is crucial, allowing the production of ATP, essential for the synthesis of NADPH, an antioxidant molecule par excellence [75].

Alzheimer’s disease (AD) is a neurodegenerative chronic and progressive disease producing irreversible deterioration of cognitive functions (memory, reasoning, and language), compromising autonomy and the ability to perform routine daily activities [76]. Oxidative stress and the changes in intracellular calcium homeostasis are common triggers of death signaling in neurons in age-related neurodegenerative diseases such as AD [77].

Changes in iron metabolism have been reported in AD. Anyhow, both in excess and deficiency, iron metabolic alterations may compromise the patient’s cognitive and behavioral state. MRI has shown in AD that the iron content in the brain is significantly increased [78], but it needs to be defined whether the accumulation of iron occurring in AD and other neurodegenerative diseases is a primary or a secondary effect of the disease.

The finding of significantly high levels of lipid peroxidation and oxidation of proteins and DNA reported in the brains of AD patients supports the hypothesis that the toxicity of Aβ could be related to its property to produce hydrogen peroxide and free radicals.

Regarding the relationship between iron and Aβ, literature data are conflicting. In particular, iron can be crucial in mediating the toxicity of Aβ and the development and progression of AD [79]. Conversely, iron has been found to promote oxidative stress and the deposition of amyloid-β protein [80]. However, some studies have demonstrated that binding the Aβ protein with iron could protect nearby neurons from oxidative stress [81,82].

In addition, antioxidant defense lacks, and mitochondrial alterations, often found in the elderly, could result in iron accumulation in AD and subsequent cell damage [83]. Early mitochondrial dysfunction and oxidative stress in AD can precede the overproduction and deposition of Aβ, which, in turn, contributes to increased oxidative stress and neurodegeneration [84,85].

Nevertheless, ferritin levels, representing iron storage, have proved to be a helpful biomarker in CSF for studying the progression of mild cognitive impairment and AD in early moderate–severe forms of the disease [86,87].

It really looks interesting that the accumulation of iron in specific brain regions shows different cognitive phenotypes. In men, excessive iron accumulation in the hippocampal region positively correlates with a worsening verbal–memory performance [88,89].

Prion diseases, such as Creutzfeldt–Jakob’s disease (sCJD), are rare, rapidly worsening brain disorders leading to dementia. CJD is a neurodegenerative disorder caused by an abnormal isoform of a cellular glycoprotein known as the prion protein. Prion disorders result from the change in prion protein (PrPC) conformation to a misfolded PrP-scrapie (PrPSc) form that accumulates in the brain parenchyma.

It is rapidly progressive and always fatal, usually leading to death within 1 year of the onset of illness. Symptoms of CJD include dementia that quickly gets worse over a few weeks or months, muscle stiffness, twitching, weakness, and problems with coordination and balance. CJD has no cure, and treatment focuses on relieving symptoms [14]. Studies have shown that sCJD-affected human brains show an increase in total iron, and there is a direct correlation between prion protein (PrP) deposition and transferrin (Tf) levels, implicating PrPSc as the underlying cause of the iron deficiency. Despite increased iron levels, diseased brains show a phenotype of neuronal iron deficiency.

Hepcidin, a key regulator of iron homeostasis, upregulated in the brain, has been demonstrated in prion diseases, including sCJD. This upregulation may contribute to iron dysregulation in prion disease-affected brains [14].

Amyotrophic lateral sclerosis (ALS), also known as motor neuron disease, is characterized by the degeneration of both upper and lower motor neurons, which leads to muscle weakness and eventual paralysis [90]. Additionally, in ALS, iron accumulation plays a role in the mechanism of neurodegeneration. The increase in iron accumulation in cortical and subcortical gray matter structures is confirmed in MND patients undergoing quantitative susceptibility mapping (QSM) or hexametric (R2*) with MRI. The primary motor cortex appears to be a cortical site where pathological iron deposition closely anticipates neuronal death in patients with ALS [91,92].

## 5. Ferroptosis and Alzheimer’s Disease in Type 2 Diabetes Mellitus

Type 2 diabetes mellitus (T2DM) is a risk factor for neurodegenerative disorders such as Alzheimer’s disease (AD) [93], resulting in cognitive decline. Hyperglycemia, insulin resistance (IR), inflammation, and oxidative stress can impair neurons, resulting in cognitive dysfunction, memory impairment, and synaptic plasticity in the hippocampus [94]. Furthermore, growing evidence shows that glycemic variability significantly impacts atherosclerosis development and cognitive function [95,96]. Considerable data suggest that, in diabetic patients, glycemic pharmacological control substantially affects complications, such as cognitive decline [97,98].

There is increasing evidence that iron and ferroptosis play a pivotal role in the pathogenesis of T2DM and its complications [99].

The relationship between iron and glucose metabolism seems reciprocal; iron affects glucose metabolism, and glucose metabolism influences several iron metabolic pathways [100]. Moreover, cellular iron overload regulates insulin production in pancreatic beta cells, critically contributing to IR, influencing hepatic metabolism, fat metabolism, and blood glucose homeostasis in multiple organs and tissues [99,101,102].

Iron increases diabetic complications, such as diabetic kidney injury [99], and likewise, ferroptosis may impair diabetes nephropathy and the renal tubule in diabetes models via the HIF-1α/HO-1 pathway [99]. Confirming this, reducing iron storage levels in vivo has improved insulin secretion and peripheral tissue insulin sensitivity, leading to better control of blood glucose and T2DM condition improvement [99].

Although the specific mechanisms regarding the relationship between iron metabolism and cognitive function in T2DM will have to be investigated, previous studies suggest that iron dysmetabolism worsens the memory function and neuronal survival in diabetics, but the mechanism responsible for this effect is mainly the worsening of insulin resistance (IR) in the CNS. Indeed, researchers suggest a link between brain iron and diabetes, particularly regarding cognitive impairment and Alzheimer’s disease, highlighted because of the common risk factors between AD and T2DM, such as IR [102]. An association between iron, adipocyte insulin resistance, and adiponectin has been demonstrated, as confirmed by a model in which iron can contribute to inducing insulin resistance. Consistent with this model, mice fed a high iron diet exhibited an iron accumulation within adipocytes and altered transcription of adipokines with insulin-sensitizing action involved in glycemic control [103].

Furthermore, brain iron accumulation has been suggested as a pathological mechanism in patients with T2DM and cognitive impairment [104]. Li J et al. demonstrated that patients with T2DM presented reduced executive function assessed through the MoCA test and elevated iron deposition in the striatum and frontal lobe. Furthermore, using radiological data from brain MRI, the authors correlated iron accumulation in the thalamus, striatum, and frontal lobe and cognitive performance in patients with T2DM, suggesting that monitoring changes in iron levels in these areas could represent a quantitative imaging marker of CNS lesions in patients with T2DM. Such an approach could be helpful in the early assessment of cognitive decline in T2DM patients [104]. As a result, ferroptosis is one of the major pathogenic factors in cognitive decline associated with T2DM. In the hippocampus of diabetic rats, the SLC40A1 (ferroportin) gene implicated in the ferroptosis signaling pathway is significantly downregulated [104,105]. In addition, iron deficiency could aggravate cognitive dysfunction through attention, memory dysfunction, and behavioral abnormalities in obese individuals [106].

## 6. Ferroptosis Inhibitors: Sperimental Studies

Since ferroptosis was shown to play a role in the neurodegenerative diseases’ pathogenesis, scientists began exploring the possibility of treating them by ferroptosis inhibitors. As reported in several clinical trials, iron chelation therapy represents a potential treatment for several neurodegenerative diseases [107].

There are only three clinically approved iron chelators: desferoxamine (DFO, Des-feral, desferrioxamine mesylate), deferiprone (DFP, L1, Ferriprox), and deferasirox (DFX, Exjade). Desferoxamine (DFO) and deferiprone (DFP) are currently being studied to counteract the oxidative stress caused by iron accumulation [7,8,9,10,11,12,13,14,15,16,17,18,19,20,21,22,23,24,25,26,27,28,29,30,31,32,33,34,35,36,37,38,39,40,41,42,43,44,45,46,47,48,49,50,51,52,53,54,55,56,57,58,59,60,61,62,63,64,65,66,67,68,69,70,71,72,73,74,75,76,77,78,79,80,81,82,83,84,85,86,87,88,89,90,91,92,93,94,95,96,97,98,99,100,101,102,103,104,105,106,107,108,109,110,111].

As preclinical and clinical trials have demonstrated, DFO and DFP may be effective treatments for AD, counteracting iron dyshomeostasis and ferroptosis [112]. In a 2-year single-blind study of 48 patients with potential AD, the treatment with the iron chelator DFE or oral placebo (lecithin) led to a significant reduction in the rate of deterioration in AD, and the mean rate of decline was twice as rapid for the no-treatment group. These results revealed the potential role of the ferroptosis inhibitor DFE in slowing the clinical progression of dementia associated with AD [113,114]

Other iron chelators (e.g., M30 and alpha-lipoic acid) and ferrostatin-1 (Fer-1) play their neuroprotective role by increasing the levels of hypoxia-inducible factor-1 alpha (HIF-1α), inhibiting neuronal cell death [115]. A recent study has shown that ferrostatin-1 (Fer-1) improves memory loss induced by the aggregation of Aβ plaques and neuronal death [116]. Moreover, iron chelators promote the expression of HIF-1α-dependent target genes, such as HO-1 [112]. Indeed, the NRF-2/HO-1 pathway inhibits ferroptosis and has neuroprotective effects [48,117,118].

The a-lipoic acid (LA) was studied in a P301S TAU transgenic mice model, proving to be an essential natural iron chelator. LA blocks the iron overload and lipid peroxidation induced by the TAU protein, reducing ROS content and increasing the expression level of GPX4 [119].

There are potential risks associated with the use of iron chelators, local and systemic, requiring cessation of therapy in up to 30% of patients. These effects include arthritis, nausea, skin rashes, ophthalmic complications, agranulocytosis, cardiac toxicity, tumor cell proliferation, and gastrointestinal symptoms such as abdominal pain, vomiting, and diarrhea. It is important to note that the benefits of iron chelation therapy usually outweigh the risks for patients with iron overload disorders. However, patients should be monitored closely for potential adverse effects, and treatment should be adjusted if necessary [120].

Several other classes of anti-ferroptosis agents may be of potential benefit.

The hydroxypyridinone derivative, CN128, has proven to be another valid agent capable of counteracting the progression of neurodegenerative diseases, particularly in clinical studies for Parkinson’s disease [121]. A double-blind, randomized phase 2 study used a hydroxyquinoline chelator, PBT2, which has been shown to minimize metal-induced protein aggregation in Huntington’s disease [122].

The N-acetylcysteine (NAC), an antioxidant and a glutathione precursor, could contribute to counteracting the evolution of AD. In AD animal models, NAC causes decreased spatial memory deficits and the loss of synaptic plasticity, reducing lipid oxidation and increasing the GSH content [105,123].

Levels of vitamin E, an anti-ferroptosis agent, decreased in the plasma, serum, and cerebrospinal fluid of AD patients [124]. However, the clinical data need to be more consistent. In a large randomized clinical trial, vitamin E exhibited clinical benefits in patients with mild to moderate AD, as measured by slowing functional decline and decreasing caregiver burden [119]. Some clinical studies have reported that high vitamin E doses slowed cognitive deterioration in AD patients [125]; other reports found that vitamin E did not slow the progression of AD [126]. In a recent clinical study [127], higher vitamin E levels were associated with decreased density of activated microglia in cortical brain regions, suggesting that vitamin E may have an anti-inflammatory effect on the microglia.

Further studies have shown that supplementation with an organic form of selenium (Se), Se-enriched yeast (Se-yeast), or selenium–methionine (Se-Met), the key regulator of GPX4-activity selenium (Se), could improve cognitive impairment [93,128]. A recent randomized controlled pilot trial found that high nutritional supplementation (24 weeks) of sodium selenate increased selenium uptake into the CNS and improved cognitive function [129].

Macromolecular approaches that offer the advantage of targeting brain iron specifically compared to small molecular chelators were also investigated. For example, a polymeric nanoparticle chelator containing a brain-targeting peptide, a glycoprotein found in the rabies virus, can intracerebrally deliver DFO in Parkinson’s disease mice. The obtained effect was a decrease in iron content and a reduction in oxidative stress levels in the substantia nigra and striatum of the mice brain. This therapeutic approach has also been shown to reduce dopaminergic neuron damage in mice without adversely affecting the other organs [130].

There are no specific clinical trials investigating the use of ferroptosis inhibitors in diabetic patients with cognitive decline. However, there are studies that suggest that ferroptosis inhibitors may have a therapeutic effect in diabetic complications, including cognitive impairment. Besides iron chelators, many studies have demonstrated the ability to signal molecules or drugs to regulate ferroptosis and ferritinophagy in the prevention and treatment of diabetes complications, including cognitive impairment. Ferroptosis and ferritinophagy are expected to become targets in the study of diabetes complications [131,132].

Moreover, ferroptosis inhibitors promise to rescue pancreatic beta-cell function and alleviate diabetes and its complications, including cognitive decline in diabetic patients [133,134].

Quercetin, a flavonoid found in many fruits and vegetables, has been shown to inhibit ferroptosis and reduce cognitive impairment in diabetic encephalopathy [135].

In addition, erythropoietin has been shown to ameliorate cognitive dysfunction in mice with type 2 diabetes mellitus via inhibiting iron overload and ferroptosis [136].

Regarding the hypoglycemic drug and ferroptosis relationship, many studies have demonstrated the drug’s ability to regulate ferroptosis [137].

Canagliflozin, a sodium–glucose cotransporter-2 inhibitor (SGLT2i) hypoglycemic drug, apart from improving the diabetic myocardial structure and its function, and reducing the risk of cardiovascular events in people with T2DM, may inhibit ferroptosis by steadily stabilizing cardiac iron and inhibiting myocardial oxidative stress [137].

Liraglutide, a glucagon-like peptide-1 (GLP-1) receptor inhibitor used to treat obesity and diabetes, plays a crucial role in ferroptosis in db/db mice. Liraglutide attenuates damage to hippocampal neurons and synaptic plasticity and restores cognitive function by inhibiting hippocampal iron death in diabetic cognitive impairment mice [137].

## 7. Conclusions

Ferroptosis remains a cell death mechanism that needs clinical and scientific investigation, to elucidate the whole short- and long-term effects of ferroptosis.

Therefore, it is urgent to identify early biomarkers that allow us to understand which brain cells and why they are more susceptible to ferroptosis and whether some chronic diseases, such as diabetes, may be mainly responsible for the pathological mechanisms of increased iron deposition and ferroptosis, especially in the brain.

Fortunately, and unfortunately, the global population is aging rapidly, and cogni-tive decline and neurodegenerative diseases, such as AD, are a societal burden. As a result, traditional hypotheses of cognitive impairment and the pathogenesis of neurodegenerative diseases and AD are evolving in new directions.

Thus, understanding iron dyshomeostasis and ferroptosis in the pathogenesis of cognitive decline and AD may also offer a promising approach to establishing new hypotheses. Given the multi-factoriality of the neurodegenerative process, using multifunctional iron chelators is a good way of development.

However, although several potential therapies modulate iron dyshomeostasis and prevent ferroptosis, the main challenge for future pharmacological research will be to further investigate the ferroptosis hypothesis in treating neurodegenerative diseases.

To identify new effective therapies in multifactorial diseases, a merely reductionist approach would provide short answers, and, therefore, only through a systems biology approach and analysis of neurodegenerative processes will it be possible to discriminate more critical players in specific pathological conditions. This approach is the only way to identify the patient’s cognitive profile and develop an accurate, personalized therapy.

In conclusion, the iron inhibitors study should yield drugs with high iron selectivity, free radical inhibitory capacity, and the ability to block protein aggregation.

Overall, while there is limited research on the specific effects of hypoglycemic drugs on ferroptosis, evidence suggests that ferroptosis inhibitors may have a therapeutic effect in preventing and treating diabetes complications such as cognitive decline. In conclusion, further research is needed to develop effective treatments targeting ferroptosis but also fully understand the molecular mechanisms underlying ferroptosis and its role in both neurodegenerative diseases and T2DM. T2DM, as chronic disease is also responsible for representing a risk factor for neurodegeneration and thus for cognitive decline.

Further research will provide practical and cost-effective treatments to fully understand ferroptosis’s role in neurodegenerative diseases and develop effective disease-modifying therapies.

## Data Availability

Not applicable.
